# Radiogenomics Map Reveals the Landscape of m6A Methylation Modification Pattern in Bladder Cancer

**DOI:** 10.3389/fimmu.2021.722642

**Published:** 2021-10-18

**Authors:** Fangdie Ye, Yun Hu, Jiahao Gao, Yingchun Liang, Yufei Liu, Yuxi Ou, Zhang Cheng, Haowen Jiang

**Affiliations:** ^1^ Department of Urology, Huashan Hospital, Fudan University, Shanghai, China; ^2^ Fudan Institute of Urology, Huashan Hospital, Fudan University, Shanghai, China; ^3^ Department of Radiology, Huashan Hospital, Fudan University, Shanghai, China; ^4^ National Clinical Research Center for Aging and Medicine, Huashan Hospital, Fudan University, Shanghai, China

**Keywords:** m6A, radiogenomics, contrast-enhanced computed tomography, immunotherapy, mutation burden

## Abstract

We aimed to develop a noninvasive radiomics approach to reveal the m6A methylation status and predict survival outcomes and therapeutic responses in patients. A total of 25 m6A regulators were selected for further analysis, we confirmed that expression level and genomic mutations rate of m6A regulators were significantly different between cancer and normal tissues. Besides, we constructed methylation modification models and explored the immune infiltration and biological pathway alteration among different models. The m6A subtypes identified in this study can effectively predict the clinical outcome of bladder cancer (including m6AClusters, geneClusters, and m6Ascore models). In addition, we observed that immune response markers such as PD1 and CTLA4 were significantly corelated with the m6Ascore. Subsequently, a total of 98 obtained digital images were processed to capture the image signature and construct image prediction models based on the m6Ascore classification using a radiomics algorithm. We constructed seven signature radiogenomics models to reveal the m6A methylation status, and the model achieved an area under curve (AUC) degree of 0.887 and 0.762 for the training and test datasets, respectively. The presented radiogenomics models, a noninvasive prediction approach that combined the radiomics signatures and genomics characteristics, displayed satisfactory effective performance for predicting survival outcomes and therapeutic responses of patients. In the future, more interdisciplinary fields concerning the combination of medicine and electronics remains to be explored.

## Introduction

The whole genome distribution of N^6^-Methyladenosine (m6A) was not revealed until 2012; it is the most common epigenetic modification of the eukaryotic transcriptome, affecting almost every process of RNA metabolism, including translation, folding, splicing, degradation, and export (1–5). m6A RNA methylation is a reversible and dynamic procedure, which is catalyzed by the m6A methyltransferase complex consisting of methyltransferases (writers), including VIRMA, WTAP, METTL3/14/15/16, RBM15, RBM15B, and ZC3H13. Among the m6A RNA methyltransferase complexes, METTL3 is the key component (6, 7). The m6A modification is removed by demethylases (erasers), including ALKBH5 and FTO (8, 9). Fourteen binding proteins act as “readers,” which specifically recognize the m6A modification and produce m6A modified RNA, including ELAVL1, FMR1, HNRNPA2B1, HNRNPC, IGF2BP1/2/3, LRPPRC, RBMX, YTHDC1/2, and YTHDF1/2/3 (10, 11). An increasing number of studies have found that m6A modification is involved in a variety of biological processes, including embryonic development disorders, tumor development, and immune cell infiltration (12–14). Notably, the imbalance of m6A modification is significantly associated with the occurrence and progression of various cancers, such as bladder cancer, pancreatic cancer, hepatocellular carcinoma, and colorectal cancer (15–18). In brief, m6A modification plays a role in carcinogenesis and tumor inhibition in diverse scenarios.

Recent progress in genetics has allowed for extensive genomics and transcriptome analyses to reveal the potential molecular mechanism underlying bladder cancer. Radiomics, another new technology, has enabled the identification of significant imaging signatures that could not be captured by the unaided eye and the exploitation of the potential characteristics of digital imaging. Radiomics can transform biomedical images into mineable quantitative characteristics, and then conduct subsequent analyses to improve the effective performance of preoperative expectation, tumor classification, prognosis prediction, and treatment response (19–21). Radiogenomics is an emerging cross-disciplinary study between radiomics and genomics, which is the simplest method used to extract high-level genomic information. In recent years, it has been extended to connect radiomics with broader biological characteristics, such as proteomics and metabolomics (22, 23). Previous studies have explored tumor gene expression, tumor mutation burden, methylation pattern, and subtypes using non-invasive digital imaging features (24–26). In addition, a combination of radiomics and genomics can contribute to improving the efficiency of clinical prediction in some cancers (27, 28). Thus, radiogenomics may help us understand the molecular phenotype of various cancers and provide real-time monitoring for the clinical management of individual patients.

Bladder cancer is the sixth most common cancer and the ninth most common cause of cancer death among males worldwide.; a highly malignant urogenital tumor characterized by hidden onset and easy misdiagnosis (29). Distinct proportions of genome subclones lead to cellular and molecular heterogeneity in this type of tumor, which affects both clinical outcomes and therapeutic responses (30, 31). Cystoscopy, as a traditional diagnostic technology, is restricted in realizing the purpose of individualized medicine because of the inability to identify genome subclones, and it is an invasive method. Thus, increasing studies have begun to focus on image processing to help predict the clinical outcomes of Bca patients. Xu X. et al. systematically retrieved the research reports on the application of radiomics in bladder cancer from 2000 to 2021, described the current blueprint of this field for researches, and comprehensively explained its pitfalls, challenges and opportunities (32). Xu S. et al. also combined with diffusion-weighted imaging (DWI) radiomics features and clinical data of transurethral resection to improve the sensitive and accuracy for the detection of muscle invasive bladder cancer (33). Therefore, it is imperative to develop a new non-invasive technology to help clinicians make correct judgement and reduce unnecessary invasive examinations.

In this study, we aimed to develop a noninvasive radiomics approach to reveal the m6A methylation status and predict survival outcomes of Bca patients. We collected the Genomic data from 716 cases of bladder cancer, and then construct the methylation modification pattern by unsupervised clustering of m6A regulators expression level. we investigated the expression of m6A regulators rather than m6A methylation itself, as the biological function of m6A methylation will alter based on genomic context. Three distinct m6A methylatin modification patterns with different tumor microenviroment were identified. In the final analysis of genomics, we identified m6A-related prognostic genes, and constructed the m6Ascore system based on the expression levels of these genes to quantify the m6A methylation status of individual samples. As for the radiomics, a total of 120 samples had complete digital images were obtained from the Cancer Imaging Archive (TCIA) database. We used a radiomics algorithm to obtain the image signature and constructed image prediction models based on the m6Ascore system classification. In brief, our findings revealed the critical role of m6A RNA methylation in bladder cancer, and we proposed a convenient method to help diagnose and predict the survival outcomes of patients with bladder cancer.

## Methods

### Data Acquisition of Bladder Cancer Samples

The transcriptome data and adjusted clinical information of bladder cancer samples were retrospectively acquired from the Gene Expression Omnibus (GEO) and The Cancer Genome Atlas (TCGA) databases. A total of 716 samples were selected for analysis, including those from the Cancer Genome Atlas Urothelial Bladder Carcinoma (TCGA-BLCA) database (n = 408) and GSE32894 dataset (n = 308). Transcriptome data and genomic mutation data of the TCGA-BLCA were obtained from the UCSC Xena database. The m6A regulators were collected based on several articles, including nine methyltransferases (writers; VIRMA, WTAP, METTL3/14/15/16, RBM15, RBM15B, and ZC3H13), two demethylases (erasers; ALKBH5 and FTO), and 14 binding proteins (readers; ELAVL1, FMR1, HNRNPA2B1, HNRNPC, IGF2BP1/2/3, LRPPRC, RBMX, YTHDC1/2, YTHDF1/2/3) (34–37). The effective clinical immunotherapy performance and digital imaging information of bladder cancer were obtained from the Cancer Imaging Archive (TCIA) database.

### Unsupervised Clustering of Twenty-Five m6A Regulators

To determine the distinct m6A modification patterns mediated by m6A regulators, unsupervised consensus clustering analysis was performed based on the expression level of the 21 identified m6A regulators (4 identified m6A regulators were excluded due to missing GSE32894 transcriptome data). Principal component analysis (PCA) was performed to determine whether each subtype was relatively independent of the others. The R package “conensusClusterPlus” was utilized to determine the cluster count, and 1000 repetitions and pltem=0.8 were executed to verify the stability of the subtype. The R package “gene set variation analysis (GSVA)” was then used to assess any differences in biological pathways among subtypes (38). The identified biological features were obtained from the Kyoto Encyclopedia of Genes and Genomes (KEGG) database (39).

### Immune Cell Infiltration and Tumor Mutation Burden Estimation

Single-sample gene set enrichment analysis (ssGSEA) is designed for analysis of a single sample that could not be processed using standard GSEA (40). The relative abundance of immune cells in bladder cancer was estimated by performing ssGSEA based on the expression levels of immune cell-related genes (41). The deconvolution algorithm “cibersort” was then employed to assess the relative abundance of 22 infiltration immune cells and the “ESTIMATE” algorithm was applied to calculate the stromal and immune abundance based on the RNA-seq data of bladder cancer. We also used the “MutSigCV” algorithm to select oncogenes with a higher mutation frequency than the background. The mutation landscapes of oncogenes and m6A regulators in TCGA-BLCA cohort were displayed using the R package “maftools.”

### Selection of m6A Prognostic Related Genes Between Diverse Subtypes

To select the m6A related genes, the empirical Bayesian function of the R package “limma” was employed to select the differentially expressed genes (DEGs) among diverse subtypes, which we termed m6A related genes. The adjusted ρ value was 0.001. We then adopted univariate Cox regression analysis to extract the m6A prognostic related genes for further analysis. To prove that m6A prognostic-related genes (MPRGs) play an important role in tumor progression, we classified tumor samples into diverse gene clusters by employing an unsupervised clustering method according to the MPRGs expression levels.

### Construction of m6Ascore Models

The above models were only based on the patient population and cannot accurately predict the m6A methylation status of an individual patient. Therefore, we designed an m6Ascore to assess the m6A modification patterns of individual samples. Based on the m6A prognostic-related gene expression level, we constructed the m6Ascore models by performing PCA. This method aimed to apply the concept of dimension reduction to transform multiple indicators into a few comprehensive indicators, whose advantage is to maintain the most important features and remove noise and insignificant features to improve data processing speed. Both principal components 1 and 2 were extracted to act as m6Ascores, the method was similar to GGI (42).


m6Ascore=Σ(PC1i+PC2i),

where i is the m6A prognostic-related gene expression level.

### Identification of Radiomics Signatures From Digital Imaging

A total of 120 image samples matched with TCGA-BLCA samples were selected from TCIA dataset, 22 samples were excluded according to specific exclusion criteria (inadequate image quality or inability of the imaging surgeon to identify the lesion area). The study eventually included 98 samples. The constructed m6Ascore model as a classifier, we extracted imaging feature from these digital images for established radiogenomic prediction models. We randomly selected 67 cases as the training dataset (46/21 = positive/negative), and the remaining 31 cases were used as an independent test dataset (21/10 = positive/negative). 98 patients were selected for a repeat region of interest (ROI) segmentation at 30 days following the initial segmentation, and this was performed by the same radiologist and an additional radiologist (6 years of experience in abdominal imaging). Then, the feature matrix was normalized. We then applied several dimensionality reduction and machine learning methods for imaging genomics model building and used the best area under the curve (AUC) value in the test group as the selection criterion to choose the best approach to construct the final model. Among them, Z-Score and Minmax normalization methods were used to normalize the data; Principal components analysis (PCA) and Pearson correlation coefficient (PCC) methods were used to pre-process the features; ANOVA, Kruskal-Wallis (KW), and Recursive feature elimination (RFE) were used to select the best features. A variety of machine learning classifiers, including SVM, LDA, logistic regression, LR-Lasso, Adaboost, Naive Bayes, and Random Forest, were used to build the radiogenomics classifier model. A total of more than a thousand models were constructed and one of them was selected as the optimal model.

Model performance was evaluated using receiver operating characteristic (ROC) curve analysis. AUC quantification was calculated. Accuracy, sensitivity, specificity, positive predictive value (PPV), and negative predictive value (NPV) were also calculated at the maximum Yorden index value of the cut-off values. We also estimated the 95% confidence intervals using 1000 samples. The above procedures were implemented using FeAture Explorer Pro (FAEPro, V 0.3.7) for Python (3.7.6).

### Statistical Analysis

All statistical analyses were performed using the R software package (version 4.0.3). Differential gene expression analysis between the diverse cohorts was performed using the R package “limma.” The correlation coefficients between the m6Ascore and the infiltration of immune cells were evaluated using Spearman coefficient analysis. The Kaplan-Meier method was used to plot survival curves of patients with bladder cancer. AUC was used to evaluate the effective performance of constructed models using R package “pROC.” The R package “RCircos” was used to determine the location of the m6A regulators and their circular sequences along the chromosomes (43).

## Results

### Landscape of m6A Regulators in Bladder Cancer

**Figure 1:**
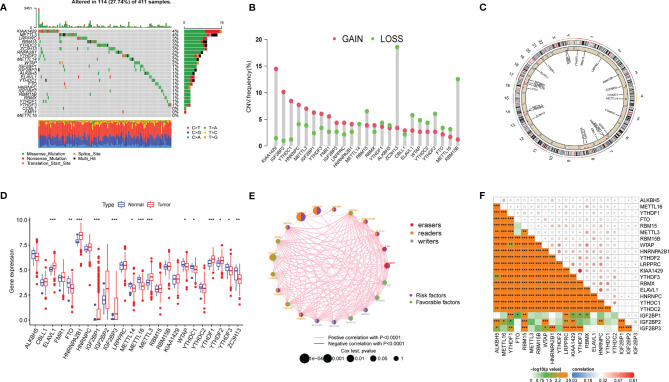
Landscape of genetic mutation and transcriptome alteration of m6A regulators in bladder cancer. **(A)** 114 of 411 samples with bladder cancer experienced genetic mutations of 25 m6a regulators, with a frequency of 27.74. the presentation of each variant types was presented by the bagplots right barplots. Each cohort represented an individual sample. **(B)** The frequency of copy number variation in GSE32894 were presented. Green dot represented deletion frequency and the red dot represented amplification frequency. The number represented the variation frequency. **(C)** The location of CNV alteration of m6A regulators on 23 chromosomes were presented by circular diagram. **(D)** The expression level of 25 m6A regulators between tumor and normal samples. Red represented tumor and blue represented normal. The asterisks represented the statistical p value (*P < 0.05; **P < 0.01; ***P < 0.001). **(E, F)** The correlation among m6a regulators, red, orange and gray represented erasers, readers and writer respectively. The size of circle represented the effect of each regulator on clinical outcomes.

A total of 25 m6A regulators were collected for further analysis, including nine methyltransferases (VIRMA, WTAP, METTL3/14/15/16, RBM15, RBM15B, and ZC3H13), two demethylases (ALKBH5 and FTO), and 14 binding proteins (ELAVL1, FMR1, HNRNPA2B1, HNRNPC, IGF2BP1/2/3, LRPPRC, RBMX, YTHDC1/2, YTHDF1/2/3). We first investigated the incidence of somatic mutations and copy number variations (CNVs) of 25 m6A regulators in bladder cancer and then summarized the gene expression distribution of m6A regulators in different samples. We found that m6A regulators were altered in 114 of 411 samples (a mutation frequency of 27.74%). The waterfall plot showed that KIAA1429 and METTL3 presented with the highest mutation frequency, mainly for missense mutations, nonsense mutations, and multiple hits, whereas METTL16 did not present as having any mutation in bladder cancer samples (**Figure 1A**). CNV frequency was found to be common in 25 m6A regulators of bladder cancer. We found that KIAA1429, ZC3H13, and RBM15B presented the highest CNV frequency (**Figure 1B**), and ZC3H13 exhibited a loss in copy number, which corresponded to the expression level of ZC3H13 in tumor samples. The location of CNV alteration of m6A regulators on 23 chromosomes is represented in a circular diagram (**Figure 1C**). We found that the expression levels of m6A regulators were significantly different between cancer and normal tissues (**Figure 1D**). The correlation and prognostic effectiveness are displayed in **Figures 1E, F**. We found that m6A regulators showed a significant correlation not only in the same function categories but also in diverse function categories, among writers, erasers, and binding proteins. The above analyses revealed a significant difference in expression alterations and genomic mutations in m6A regulators between cancer and normal tissues, which suggested that the m6A regulators played a vital role in tumor development.

### m6AClusters Mediated by m6A Regulators

**Figure 2:**
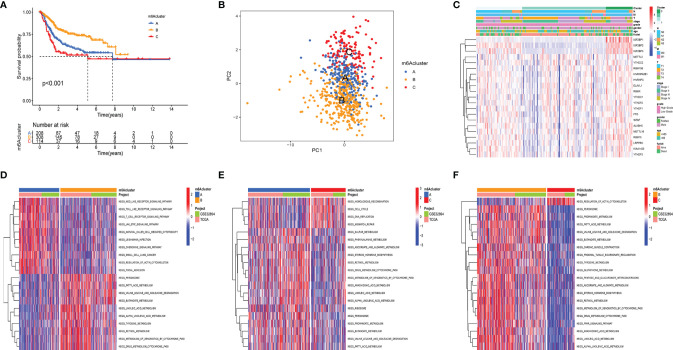
m6a methylation modification clusters and relevant biological characteristics. **(A)** Kaplan-Meier analyses for the three m6A modification clusters based on 716 patients with bladder cancer from metacohorts (TCGA-BLCA and GSE32894) Log-rank p value <0.001. **(B)** Principal component analysis verified the sample distribution of the m6aClusters. **(C)** The heatmap was performed to display the expression level of 21 m6a regulators in distinct subtypes. The m6Acluster, N stage, M stage, T stage, tumor stage, tumor grade, gender, age, and survival status were utilized as patients’ annotations. Red/green represented high/low expression of regulators **(D–F)** the GSVA investigated the biological process alteration among the three m6aClustersthe. The heatmap displayed the biological processes among each cluster. The meta cohorts (GES32894 and TCGA-BLCA) were utilized as sample annotations.

The clinical and transcriptome data of the TCGA-BLCA cohorts and GSE32894 were integrated into one meta-cohort for further analysis. The prognostic value of 21 m6A regulators was demonstrated using Kaplan–Meier (K-M) survival curves (**Supplementary Figure 1**). The results displayed that m6A regulators were significantly correlated with patients’ clinical outcome. Then, unsupervised consensus clustering analysis was used to classify patients into diverse subtypes based on the expression level of the m6A regulators. Three m6AClusters were identified, including 208 samples in m6ACluster A, 308 samples in m6ACluster B, and 114 samples in m6ACluster C. Among these m6AClusters, m6ACluster B presented a significant survival advantage, while m6ACluster C exhibited the worst clinical outcome in the meta-cohort (**Figure 2A**). The PCA results also proved that the three subtypes were relatively independent of each other (**Figure 2B**). The heatmap shows the differential expression levels of m6A regulators among m6AClusters (**Figure 2C**). The expression of IGF2BP1/2/3 was significantly reduced in m6ACluster B, which revealed that IGFBP1/2/3 may play a vital role in cancer development. GSVA was employed to investigate the biological process alteration among the three m6AClusters. The result revealed that m6ACluster A was characterized by immune activation, which enriched in toll like receptor signaling pathway, nod like receptor signaling pathway, T cell receptor signaling pathway and chemokine signaling pathway. m6ACluster B was characterized by alteration of metabolism, and m6ACluster C was significantly enriched in the cell proliferation pathway (**Figures 2D–F**).

### Selection of m6A Prognostic Related Genes (MPRGs) Between Diverse Subtypes

**Figure 3:**
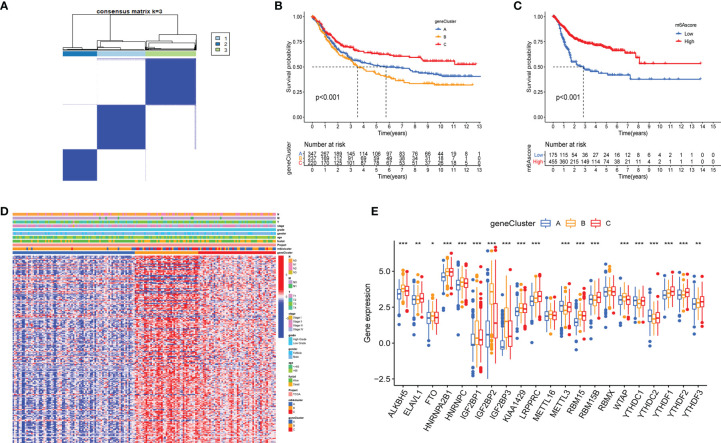
Construction of geneClusters and m6Ascore models. **(A)** Consensus matrix displayed three major clusters. **(B, C)** Kaplan-Meier analyses for the geneClusters and m6Ascore models based on 716 patients with bladder represented significant difference, Log-rank p value <0.001. **(D)** The heatmap represented significant difference in transcriptome aspect among distinct geneClusters. The N stage, M stage, T stage, tumor stage, tumor grade, gender, age, survival status, m6Acluster and geneClusters were utilized as patients’ annotations. Red/blue represented high/low expression of regulators. **(E)** The expression level of 21 m6A regulators among geneClusters. blue represented geneCluster A; yellow represented geneCluster B and red represented geneCluster C. The asterisks represented the statistical p value (*P < 0.05; **P < 0.01; ***P < 0.001).

Although the identified m6AClusters can effectively distinguish the clinical outcomes of patients with bladder cancer, the potential genetic mutations and transcriptome alterations in these subtypes are not clear. We investigated the potential m6A related genes among the diverse m6AClusters to reflect their potential effective mechanism in bladder cancer. The R package “limma” was used to select the DEGs among diverse m6AClusters, including 229 genes. (**Supplementary Figure 2**). Gene ontology (GO) enrichment analysis of these m6A related DEGs, which is summarized in **Supplementary Figure 2**, revealed that the enrichment of biological processes is significantly associated with cell proliferation and energy metabolism. These results further indicated that m6A related genes were significantly associated with tumor development. A total of 213 MPRGs were selected using univariate Cox regression analysis (**Table S1**). We applied an unsupervised clustering method to classify patients into three subtypes: gene cluster A, gene cluster B, and gene cluster C (**Figure 3A**). The K-M survival method demonstrated a significant difference among these gene subtypes; GeneCluster C presented a significant survival advantage, while GeneCluster B had the worst clinical outcome in the meta-cohort (**Figure 3B**). The expression levels of twenty-five m6A regulators in distinct gene clusters were compared, and it was observed that the expression levels of twenty-five m6A regulators were significantly different among each gene cluster (**Figure 3E**). Furthermore, the heatmap also showed significant difference among each gene clusters in the entire transcriptome, suggesting that genomic subgroup can distinguish patients from distinct m6A methylation status. (**Figure 3D**).

### Construction of m6Ascore Models

**Figure 4:**
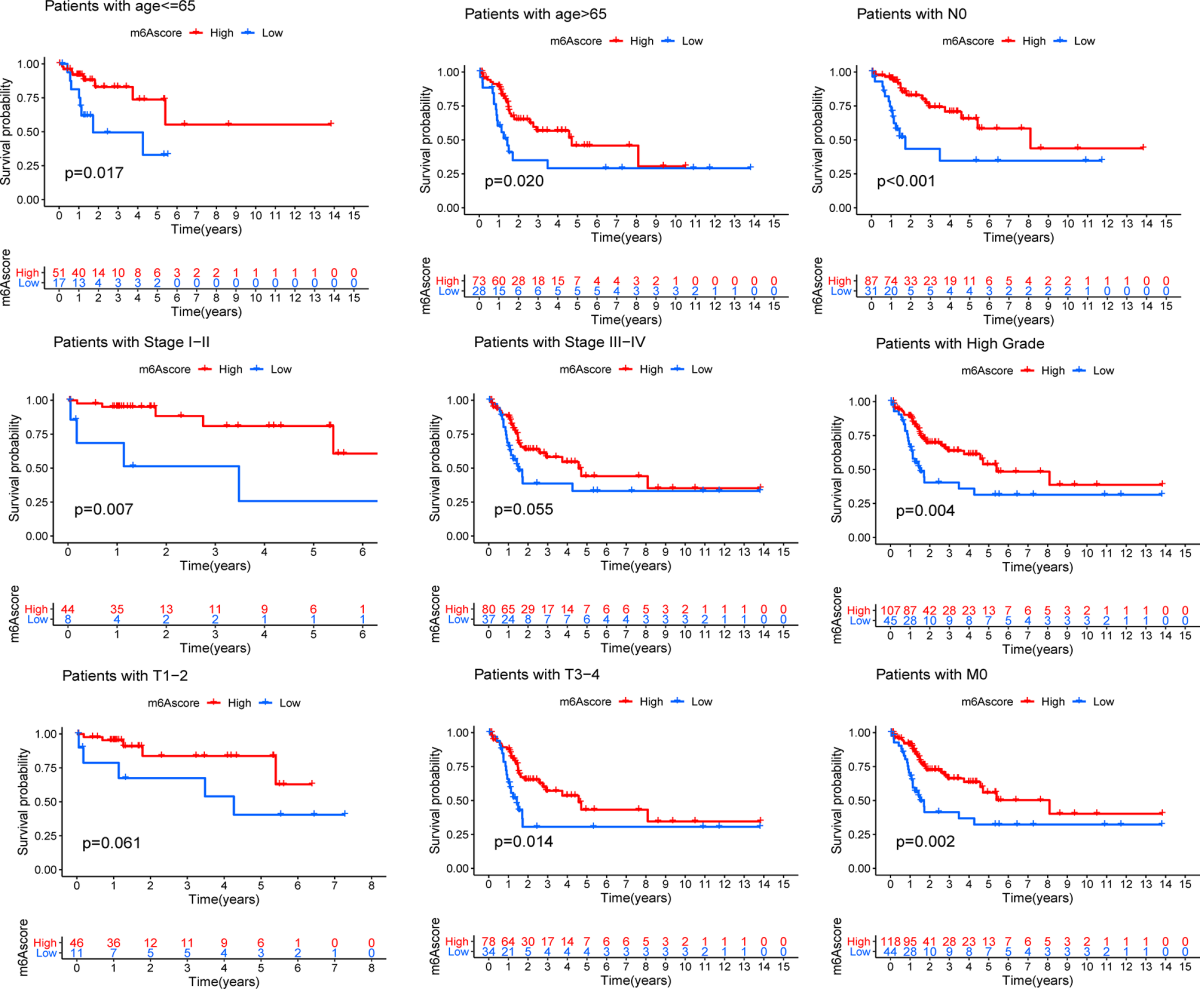
Kaplan-Meier analyses for the m6Ascore models based on the distinct clinical pathology. ns > 0.05.

Although the results of this study can predict the survival status of Bca patients, these investigations were based on the patient population and hence cannot accurately predict the m6A methylation status of an individual patient. Therefore, we constructed the m6Ascore models by performing PCA according to the m6A prognostic-related gene expression levels, which could qualify the m6A methylation status of individual patients with bladder cancer. The m6Ascore as well as PCA1 and PCA2 score were displayed in **Table S2**. The K-M survival method demonstrated a significant difference between the m6Ascore groups; patients with a high m6Ascore exhibited a significant survival advantage, while the patients with low m6Ascore had the worst clinical outcomes in the meta-cohort (**Figure 3C**). At the same time, patients were classified into different cohorts according to their clinical characteristics (age, N stage, clinical stage, grade, T stage and M stage), and it was found that the m6Ascore exhibited good predictive performance in the different clinical cohorts, including patients categorized by age and cancer stage (**Figure 4**). The bar plots and box plots showed that m6Ascore could help distinguish between different clinical pathologies. The patients with a low m6Ascore presented with a higher degree of malignancy in tumor samples, which further verified that the m6Ascore had good predictive performance, predicting not only the clinical outcomes but also the clinical traits (**Supplementary Figure 3**).

### Immune Cell Infiltration in m6AClusters

**Figure 5:**
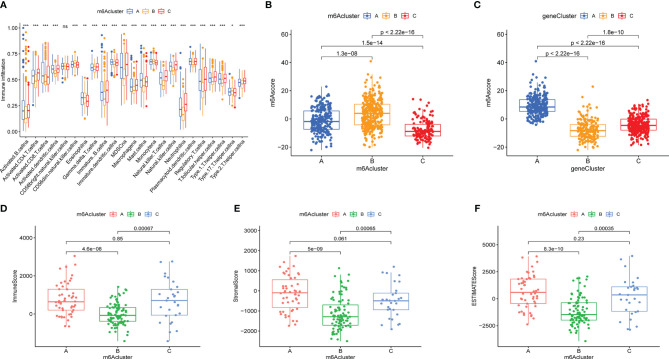
The immune cell infiltration characteristics and the relationship between m6Ascore and m6A subtypes. **(A)** The content of each tumor microenvironment infiltrating cells in three m6Aclusters. The asterisks represented the statistical p value (*P < 0.05; **P < 0.01; ***P < 0.001; ns > 0.05). **(B, C)** The correlation between m6Ascore and m6A subtypes were evaluated by Kruskal-Waillis test. The thick line of box represented the median value. **(D–F)** The immuneScore, stromalScore and ESTIMATEScore of these m6Aclusters were plotted.

To explore the immune cell infiltration alteration underlying the three diverse m6AClusters, a box plot of the relative content of immune cells among the distinct subtypes was plotted by performing ssGSEA (**Figure 5A**). The results demonstrated that almost all immune cells were reduction in m6ACluster B, and increased in m6ACluster A and m6ACluster C. The immune cell infiltration was also assessed using the “ESTIMATE” algorism, which was consistent with the results of ssGESA that the immune score was the lowest in the m6ACluster B. (**Figure 5D**). Then stromal purity (StromalScore) and tumor cell purity (ESTIMATEScore) in the three m6AClusters were also evaluated, which displayed that StromalScore and ESTIMATEScore were increased in m6ACluster A and m6ACluster C, decrease in m6ACluster B (**Figures 5E, F**).

### Prognostic Value of m6Ascore

**Figure 6:**
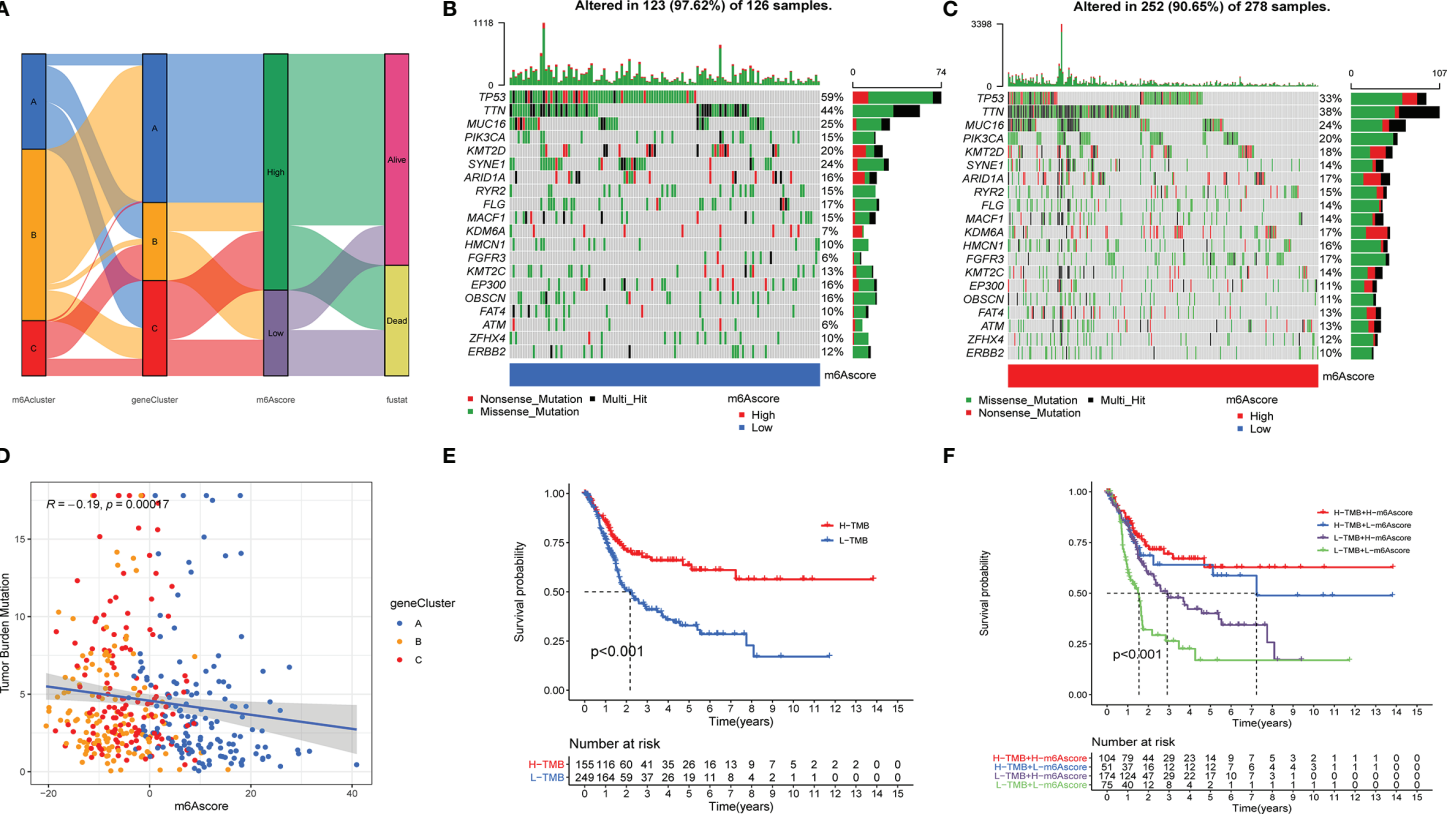
The relationship of m6Ascore and tumor burden mutation. **(A)**. The SamKey diagram of m6A methylation modification in groups with distinct m6Aclusters, geneClusters, m6Ascore, and clinical outcomes. **(B, C)** The landscape of tumor somatic mutation in TCGA-BLCA displayed by low **(B)** and high m6Ascore **(C)**. Each column represented individual patients. The upper barplot displayed tumor mutation burden. **(D)** The correlation among m6Ascore, tumor burden mutation and geneClusters, blue, yellow and red point represents geneCluster A, B, C respectively. **(E, F)** the Kaplan-Meier analyses for The TMB subtypes and TMB-m6Ascore subtypes represented significant difference, Log-rank p value <0.001.

To visualize the relationship between the above models, the clinical pathology and clinical outcomes of the Sankey diagram were plotted (**Figure 6A** and **Suplementary Figure 4**). The Kruskal-Wallis test was used to better reveal the correlation between the above models and the m6AScore. **Figure 5B** shows that m6ACluster B presented the highest median m6Ascore, and **Figure 5C** shows that GeneCluster B presented the lowest median m6Ascore. m6ACluster B and GeneCluster B exhibited a significant survival advantage and poor clinical outcomes, respectively, which is consistent with the prediction made using the m6Ascore. Thus, these results confirmed that m6Ascore could qualify the m6A methylation status of individual patients with bladder cancer.

We further analyzed the relationship between m6Ascore and tumor mutational burden (TMB). The scatter diagram showed that m6Ascore was significantly associated with the TMB (**Figure 6D**), and the waterfall plot also demonstrated that patients with low m6Ascore presented more extensive TMB than did the patients with high m6Ascore (**Figures 6B, C**). K-M analysis revealed that the patients with H-TMB exhibited a significant survival advantage, and the patients with L-TMB had the worst clinical outcomes (**Figure 6E**). To further accurately qualify the clinical status of patients with bladder cancer, we combined TMB and m6Ascore to predict the clinical outcomes of each patient. K-M analysis showed that patients with H-TMB and high m6Ascore exhibited a significant survival advantage, and patients with L-TMB and low m6Ascore had the worst clinical outcomes (**Figure 6F**).

Besides, we observed that immune response markers such as PD1 and CTLA4 were significantly associated with the m6Ascore, while patients with high m6Ascore presented lower expression levels of PD1 and CTLA4 (**Supplementary Figures 3C, D**). So, we investigated whether the m6Ascore could predict a patients’ response to immunotherapy treatment. Our results showed that the m6Ascore predicts that patients, who express CTLA4+/PD1-, respond to immunotherapy (**Supplementary Figure 3E**).

### Construction of Optimal Radiomics Signatures

**Figure 7:**
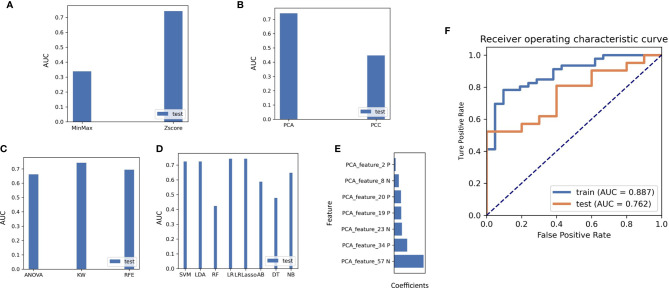
**(A) **The effect on the AUC values of the radiomics model when different methods of normalizing the imaging data are used. **(B)** Effect of the choice of feature reduction method on the AUC value of the model. **(C)** Impact of feature selection methods on classifier AUC values during radiogenomics model building. **(D)** Comparison of different machine learning modelling approaches on classifier performance in the test set, including SVM, LDA, RF, LR, LRlassp, AB, DT, NB, among which LRLasso achieved best performance. **(E)** Weighting coefficients of the extracted features in the final radiogenomics model. **(F)** Results of the AUC values of the best classifier model in the training and test groups.

A total of more than a thousand models had been constructed *via* combination of several methods from each step, and the detail information of this models were displayed in **Table S3**. Using the classifier AUC values of the test group as the selection criteria for the best model, we can find that the best predictive efficacy of the imaging genomics model was achieved by a machine learning approach built with the Z-SCORE method for data normalization, the PCA method for feature pre-processing, the KW method for dimensionality reduction, and application of the LASSO-constrained logistic regression method. A comparison of the AUC values for different data pre-processing and modeling methods is shown in **Figures 7A–D**. Seven features were identified as optimal for radiomics, and the process is shown in the **Supplementary Word**. Based on these seven features, we found that the model obtained the highest AUC value for the validation dataset. At this point, the model achieved an AUC degree of 0.887 and 0.762 for the training and test datasets, respectively. The ROC curves are shown in **Figure 7F**. The selected features are shown in **Figure 7E**.

The calculation formula of the radiomics model can be seen in follow: Radscore=0.040*PCA_Feature_2 - 0.103*PCA_Feature_8 + 0.176*PCA_Feature_19 + 0.175*PCA_Feature_20 - 0.183*PCA_Feature_23 + 0.297*PCA_Feature_34 - 0.315*PCA_Feature_57. The characteristics of PCA_feature was displayed in **Table S4**.

## Discussion

With the constant advancement in gene sequencing technology which focuses on the function and influence of reversible RNA modification, the concept of epigenetic transcriptomics has gradually gained the attention of researchers. Due to the complexity of m6A level detection (m6A MeRIP and m6A-seq), several studies have reported alternative approaches to identify the genetic mutation of m6A regulators, and evaluated the relationship between the m6A methylation modification pattern and cancer diseases. Abnormal levels of m6A regulators have exhibited a predictive benefit in many types of cancer, such as bladder cancer (44, 45), renal clear cell carcinoma (46), prostate cancer (47, 48), and breast cancer (49). Xie et al. reported that the interaction between IGF2BP1 and circPTPRA suppresses bladder cancer progression (50). Yang et al. found that METTL3 and CDCP1 are upregulated in bladder cancer and are associated with the progression of bladder cancer (51). Jin et al. explored that m6A writer METTL3 and eraser ALKBH5 regulator cell adhesion *via* embellishing ITGA6 expression in bladder cancer (44). Xie et al. demonstrated that METTL3/YTHDF2 m6A axis degraded the mRNA of tumor suppressors SETD7 and KLF4, contributing to the progression of bladder cancer (52). The differential expression of regulators involved in diverse tumor types provided us with a clue that the maladjustment of m6A regulators at the tissue level is complicated. Therefore, further studies on m6A regulators are required to explore the regulatory mechanism underlying m6A RNA modification in bladder cancer.

In this study, we classified bladder cancer samples into three distinct m6A methylation clusters, which can effectively predict the clinical outcome of bladder cancer, namely m6ACluster A, m6ACluster B, and m6ACluster C. We observed significant differences in immunocytes among the three m6A methylation clusters. Although the m6ACluster A and m6Acluster C were characterized by immune activation, which represented by high infiltration of activated CD8 T cells, activated B cells, nature killer cells, revealed a hot immune microenviroment, the activation of the stromal cells prevented the penetration of immune cells into the parenchyma of tumors. Therefore, it was no surprised that m6ACluster A and m6ACluster C has a poor clinical prognosis than m6Acluster B.

Beside, DEGs among thses three m6AClusters were regarded as m6A methylation-related genes and might be indirectly or directly alter m6A methylation status. Then we selected m6A prognostic-related DEGs by univariate Cox regression analysis. Three transcriptome clusters were constructed according to the m6A prognostic-related factors, which were significantly correlated with clinical outcomes. The transcriptome clusters further confirmed that these genes were related to m6A methylation status and the progression of bladder cancer.

Considering the intertumoral heterogeneity, we further constructed a quantitative model termed “m6Ascore” to qualify the m6A methylation status of individual samples. in order to accurately guide the treatment of individual patients. Further analysis found that m6Ascore not only can be used to predict the clinical prognosis of patients, but also can accurately distinguish between different clinical pathlogys. In addition, immune response markers such as PD1 and CTLA4 were significantly related to the m6Ascore, which indicated that m6Ascore had the ability to assess the effective performance of immunotherapy treatment. Moreover, when combined with TMB, m6Ascore has more accurate prediction performance. Therefore, these results verified m6A models may be used in clinical evaluations and targeted therapeutic schedules.

However, genomic prediction models are cumbersome and invasive, which are not conductive to auxiliary diagnosis by clinicians. Thus, we attempt to find a convenient approach to predict patients’ genetic subtype for making the appropriate clinical diagnosis. In the era of biological information digitization, radiomics analysis has been used to capture quantitative signatures from digital images that were related to the clinical pathology or molecular characteristics of patients (53). In this context, an increasing number of studies have focused on presenting genomic information on bladder cancer *via* digital imaging. For example, Wu et al. constructed a radiomics nomogram for preoperative prediction of lymph node metastasis in bladder cancer (54). Zheng et al. evaluated the muscular invasiveness of bladder cancer by performing radiomics analysis, and the AUC was as high as 0.913 (55). In this study, AUC values as the selection criteria for identifying the best model, several methods were conducted in this researched to extract the imaging features and construct radiogenomic models. It was found that the best predictive efficacy of the imaging genomics model was achieved by a machine learning approach built with the Z-SCORE method for data normalization, then the PCA method for feature pre-processing, subsequently, the KW method for dimensionality reduction, finally application of the LASSO-constrained logistic regression method for building radiogenomics classifier models. Seven features were identified as optimal for radiogenomic model. In brief, we constructed a non-invasive radiogenomic model to predict the m6a methylation status of individual patients, which may be beneficial for clinician to carry out individualized medical treatment, such as the combination of targeting m6A regulators and immunotherapy. Subsequent studies will further explore how to alter the m6a status of patients to improve the clinical prognosis.

## Conclusion

The presented radiogenomics model, a noninvasive prediction approach that combined the radiomics signatures and genomics characteristics, displayed satisfactory effective performance for predicting survival outcomes and therapeutic responses of patients with bladder cancer. More interdisciplinary studies that combine medicine and electronic fields need to be explored.

## Data Availability statement

Publicly available datasets were analyzed in this study. This data can be found here: https://www.jianguoyun.com/p/DQD2zAcQh7PNCRirjPwD and https://portal.gdc.cancer.gov/, with the accession number TCGA-BLCA; https://www.ncbi.nlm.nih.gov/geo/, with the accession number GSE32894.

## Author Contributions

FY, YH, and JG contributed equally to this work. FY and JG designed and conceptualized the study. HJ supervised the study. All authors contributed toward data collection and analysis.

## Funding

This study was supported by the National Natural Science Foundation of China (Grant Numbers: 81872102).

## Conflict of Interest

The authors declare that this research was conducted in the absence of any commercial or financial relationships that could be construed as a potential conflict of interest.

## Publisher’s Note

All claims expressed in this article are solely those of the authors and do not necessarily represent those of their affiliated organizations, or those of the publisher, the editors and the reviewers. Any product that may be evaluated in this article, or claim that may be made by its manufacturer, is not guaranteed or endorsed by the publisher.

## Supplementary Material

Supplementary Figure S1 The prognostic value of twenty-five m6a regulators were displayed *via* K-M survival curves.

Supplementary Figure S2 The bar and box chart of m6A score in groups with distinct tumor grade, tumor stage, T stage, and clinical outcomes.

Supplementary Figure S3
**(A)** The venn diagram displayed 229 overlapping genes were selected among m6Aclusers **(B)** The top 10 enriched GO terms including (BP, CC and MF). **(C, D)** validation of the CTLA4 and the PD-L1 expression level in low and high-m6Ascore group. **(E)** Validation of the immune response in low and high-m6Ascore group.

Supplementary Figure S4 The SamKey diagram of m6A methylation modification in groups with distinct m6Aclusters, geneClusters, m6Ascore, and clinical outcomes.
