# Supplementary material for: Radiogenomics Map Reveals the Landscape of m6A Methylation Modification Pattern in Bladder Cancer
Source: Front Immunol. 2021 Oct 18;12:722642. doi: 10.3389/fimmu.2021.722642 (PMC8559436; doi:10.3389/fimmu.2021.722642)
Supplement: Supplementary Figure S1 — The prognostic value of twenty-five m6a regulators were displayed via K-M survival curves. [file DataSheet_1.zip › supplementary method.docx]

Radiogenomics map reveals the landscape of m6A methylation modification pattern in bladder cancer

**Fangdie Ye^1,2 †^, Yun Hu^1,2 †^, Jiahao Gao^4†^, Yingchun Liang^1,2^, Yufei Liu^1,2^, Yuxi Ou^1,2^, Zhang Cheng^1,2^, Haowen Jiang^1,2,3*^**

^1^Department of Urology, Huashan Hospital, Fudan University, Shanghai 200040, China

^2^Fudan Institute of Urology, Huashan Hospital, Fudan University, Shanghai 200040, China

^3^National Clinical Research Center for Aging and Medicine, Huashan Hospital, Fudan University, China

^4^Department of Radiology, Huashan Hospital, Fudan University, Shanghai, China

*** Correspondence:**Corresponding Author: Prof. Haowen Jiang
[urology_hs@163.com](mailto:urology_hs@163.com)

**^†^**These authors contributed equally to this work

**Supplementary methods**

**1.Data sources and preprocessing**

Transcriptome data and clinical information were downloaded from the Gene Expression Omnibus (<https://www.ncbi.nlm.nih.gov/geo/>) and The Cancer Genome Atlas (<https://portal.gdc.cancer.gov/repository>).

The simple nucleotide variation and copy number variation were only downloaded from The Cancer Genome Atlas (<https://portal.gdc.cancer.gov/repository>).

The digital images and immunotherapy information were downloaded from The Cancer Imaging Archive(<https://www.cancerimagingarchive.net/access-data/>). The patient information we collected in this database has been consistent with that in TCGA database.

**2. Generation of m6A prognostic related genes and m6Ascore computation**

The generation of m6A prognostic related genes was performed as follows. First, each differentially expressed genes (DEGs) among the three m6Acluster (A, B, and C) were determined as m6A related genes. Then univariate Cox regression analysis was adopted to extract the m6A prognostic related genes for further analysis. To prove that m6A prognostic-related genes (MPRGs) play an important role in tumor progression, the unsupervised clustering method (ConsensusClusterPlus) for analysis of m6A prognostic related genes was used to classify patients into three groups for further analysis. Principal components analysis (PCA) was conducted to obtain the prognostic value of the signature score for each gene pattern, we applied a method similar to GGI to calculate the m6Ascore of each patient: m6Ascore = ∑(PC1i+PC2i),

where i is the m6A prognostic-related gene expression level.

**3.Bridge between radiomics and genomics**

A total of 120 samples included image data as well as transcriptome and clinical data. The image data were selected from TCIA dataset, the transcriptome and clinical data were obtained from TCGA database (these samples are part of the data we used to analyze the m6A status, so we use it as a bridge between radiomics and genomics)  . 22 samples were excluded according to specific exclusion criteria (inadequate image quality or inability of the imaging surgeon to identify the lesion area). The study eventually included 98 samples.

**4. radiogenomic eigenvector processing and model construction**

For each eigenvector, the mean and standard deviation were calculated. Each eigenvector was subtracted from the mean and divided by the standard deviation. After normalisation, each vector had a zero centre and a unit standard deviation. Due to the high dimensionality of the feature space, we applied principal component analysis (PCA) to the feature matrix. Eigenvectors of the transformed eigenmatrices are independent of each other.Before creating the model, features were selected using Kruskal Wallis, a commonly applied method to explore the salient features corresponding to the labels. Calculating F-value to evaluate the relationship between features and labels. We rank the features according to their corresponding F-value and select top 7 features based on validation performance. LASSO constrained logistic regression is used as our classifier. A Logistic Regression with LASSO Constraints is a linear classifier based on Logistic Regression. The L1 paradigm is added to the final loss function and the weights are constrained to make the features sparse. To determine the hyperparameters of the model (e.g. number of features), a 10-fold cross-validation was applied to the training dataset. Hyperparameters were set based on the performance of the model on the validation dataset.

The constructed m6Ascore model as a classifier, we extracted imaging feature from these digital images for established radiogenomic prediction models. We randomly selected 67 cases as the training dataset (46/21 = positive/negative), and the remaining 31 cases were used as an independent test dataset (21/10 = positive/negative). 98 patients were selected for a repeat region of interest (ROI) segmentation at 30 days following the initial segmentation, and this was performed by the same radiologist and an additional radiologist (6 years of experience in abdominal imaging). Then, the feature matrix was normalized. We then applied several dimensionality reduction and machine learning methods for imaging genomics model building and used the best area under the curve (AUC) value in the test group as the selection criterion to choose the best approach to construct the final model. Among them, Z-Score and Minmax normalization methods were used to normalize the data; Principal components analysis (PCA) and Pearson correlation coefficient (PCC) methods were used to pre-process the features; ANOVA, Kruskal-Wallis (KW), and Recursive feature elimination (RFE) were used to select the best features. A variety of machine learning classifiers, including SVM, LDA, logistic regression, LR-Lasso, Adaboost, Naive Bayes, and Random Forest, were used to build the radiogenomics classifier model. A total of more than a thousand models were constructed and one of them was selected as the optimal model.

Model performance was evaluated using receiver operating characteristic (ROC) curve analysis. AUC quantification was calculated. Accuracy, sensitivity, specificity, positive predictive value (PPV), and negative predictive value (NPV) were also calculated at the maximum Yorden index value of the cut-off values. We also estimated the 95% confidence intervals using 1000 samples. The above procedures were implemented using FeAture Explorer Pro (FAEPro, V 0.3.7) for Python (3.7.6).

**5. Other R package functions**

R package “RCircos” was conducted to visualize the location of the m6A regulators and their circular sequences along the chromosomes.

R package “limma” was employed to select the Differential expression genes.

R package “maftools” was performed to visualize the mutation landscapes.

R packages including “igraph”, “psych”, “resharpe2”, “RColorbrewer” were used to construct m6A network diagram.

R package “ConsensusClusterPlus” was employed to constructed distinct m6A subtypes.

R packages “GSVA”, “GSEABase” were conducted to visualize significant differential pathway, and displayed the immune activation among distinct subtypes.
