# Supplementary figures and images for: Radiogenomics Map Reveals the Landscape of m6A Methylation Modification Pattern in Bladder Cancer
Source: Front Immunol. 2021 Oct 18;12:722642. doi: 10.3389/fimmu.2021.722642 (PMC8559436; doi:10.3389/fimmu.2021.722642)

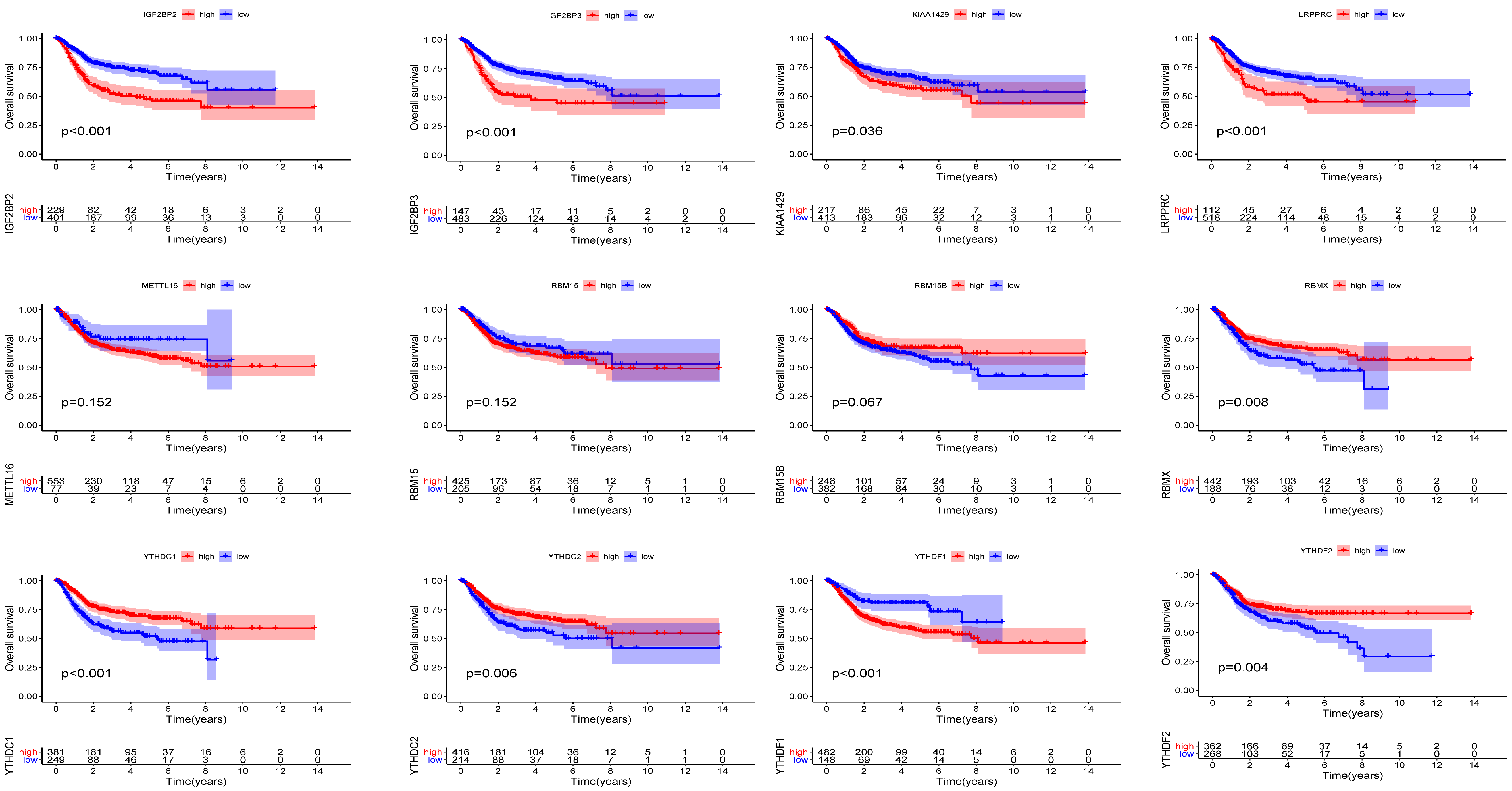

Supplement: Supplementary Figure S1 — The prognostic value of twenty-five m6a regulators were displayed via K-M survival curves. [file DataSheet_1.zip › S1(1).tif]

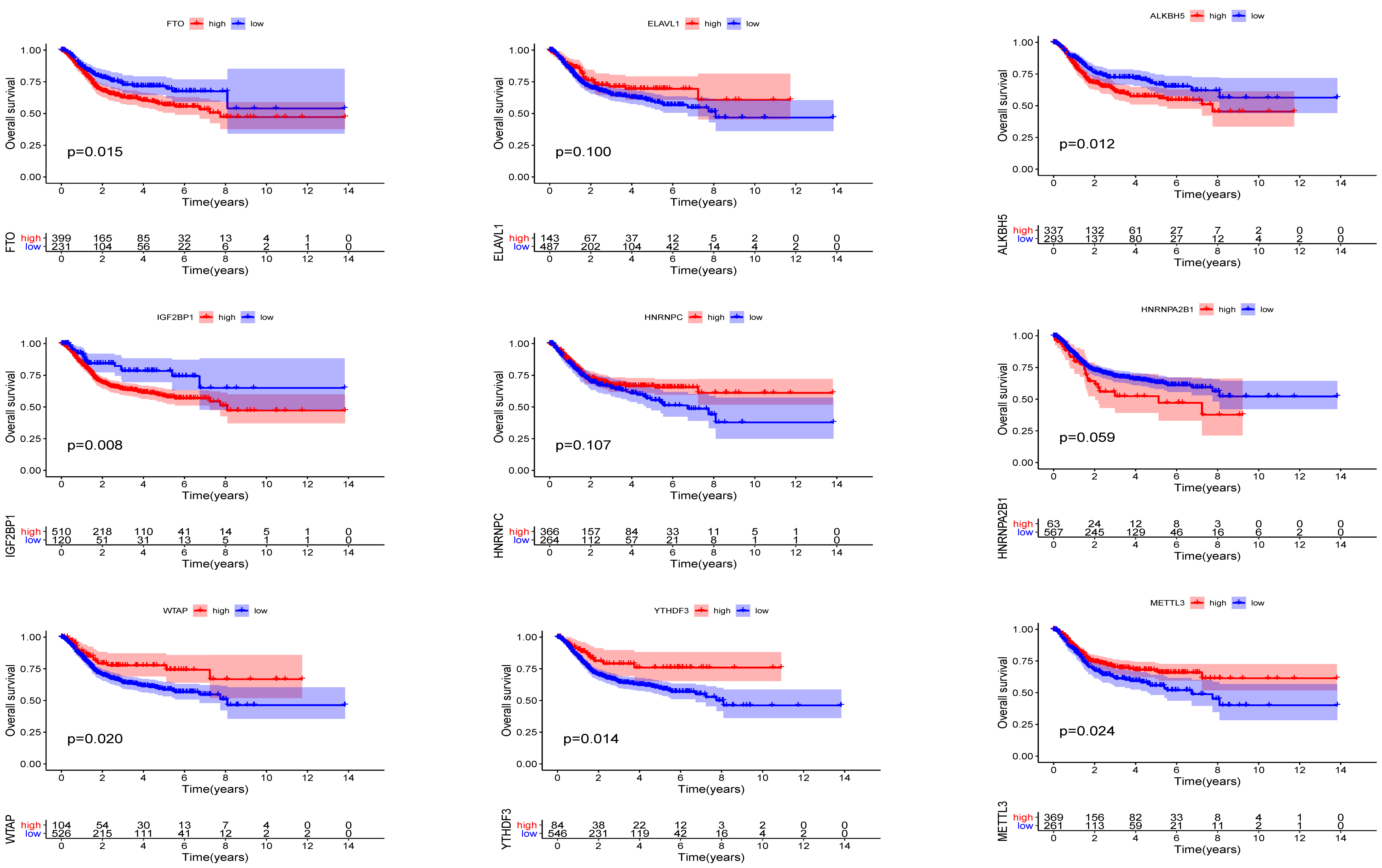

Supplement: Supplementary Figure S1 — The prognostic value of twenty-five m6a regulators were displayed via K-M survival curves. [file DataSheet_1.zip › S1(2).tif]

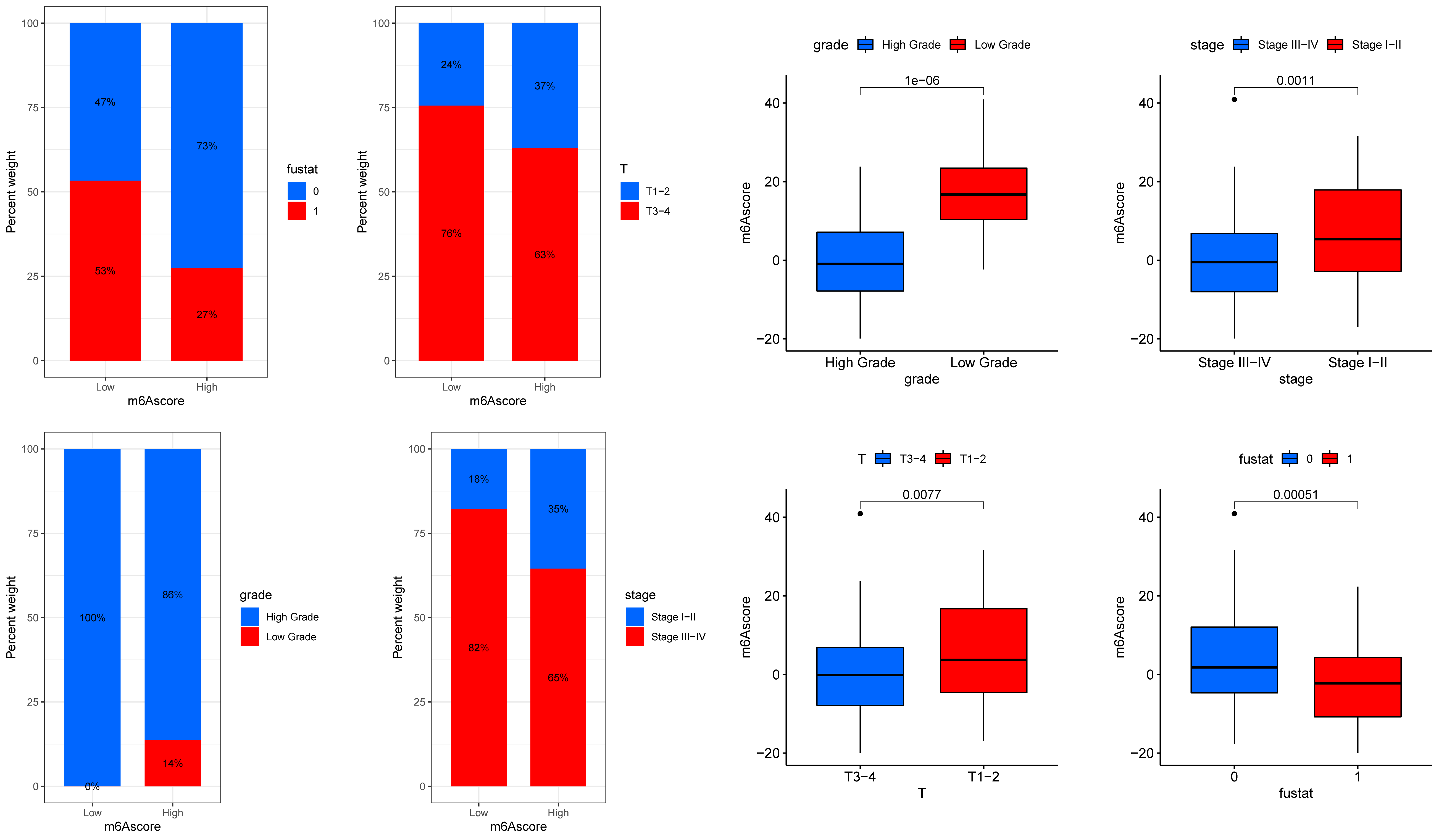

Supplement: Supplementary Figure S1 — The prognostic value of twenty-five m6a regulators were displayed via K-M survival curves. [file DataSheet_1.zip › S2.tif]

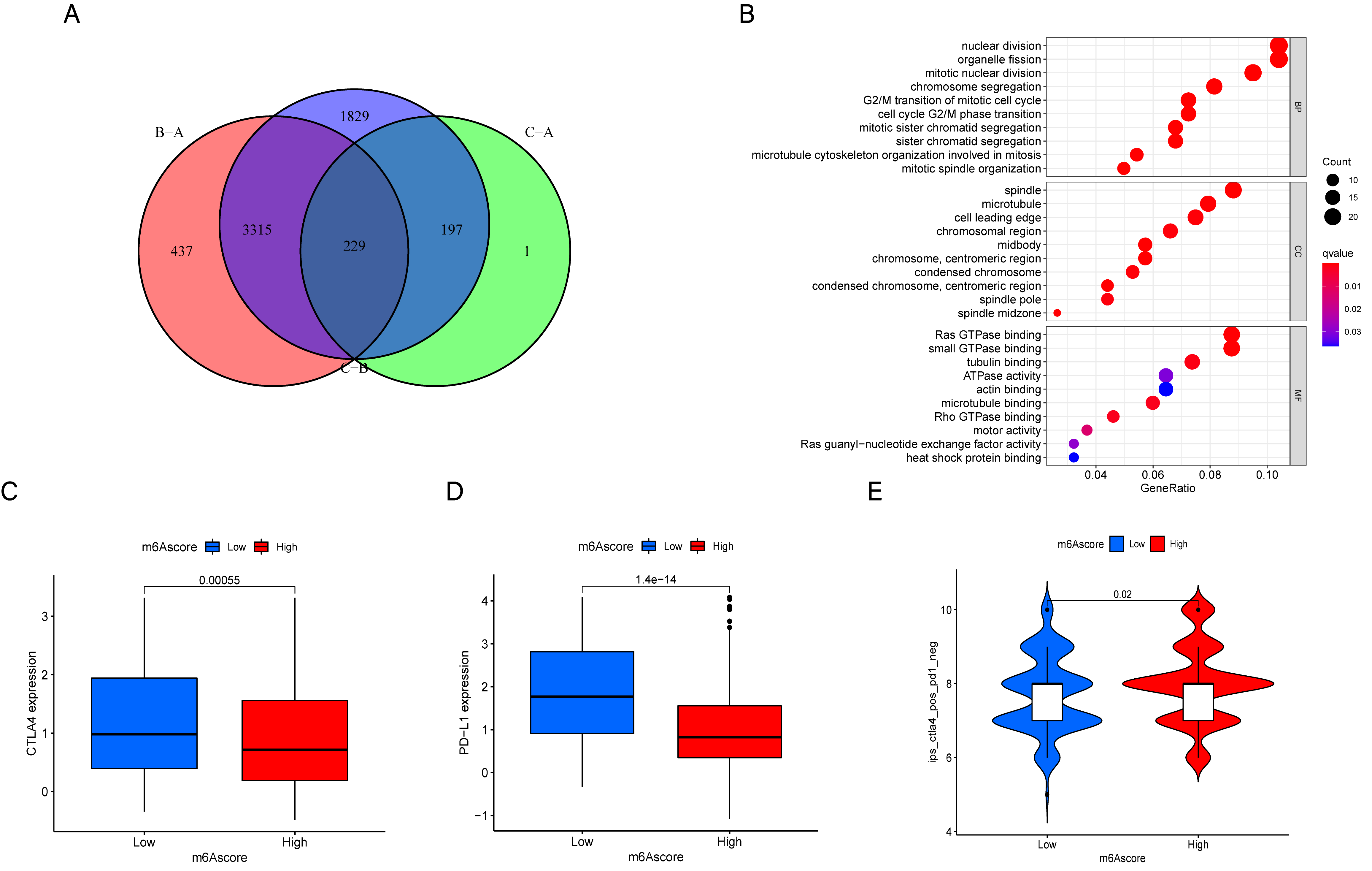

Supplement: Supplementary Figure S1 — The prognostic value of twenty-five m6a regulators were displayed via K-M survival curves. [file DataSheet_1.zip › S3.tif]

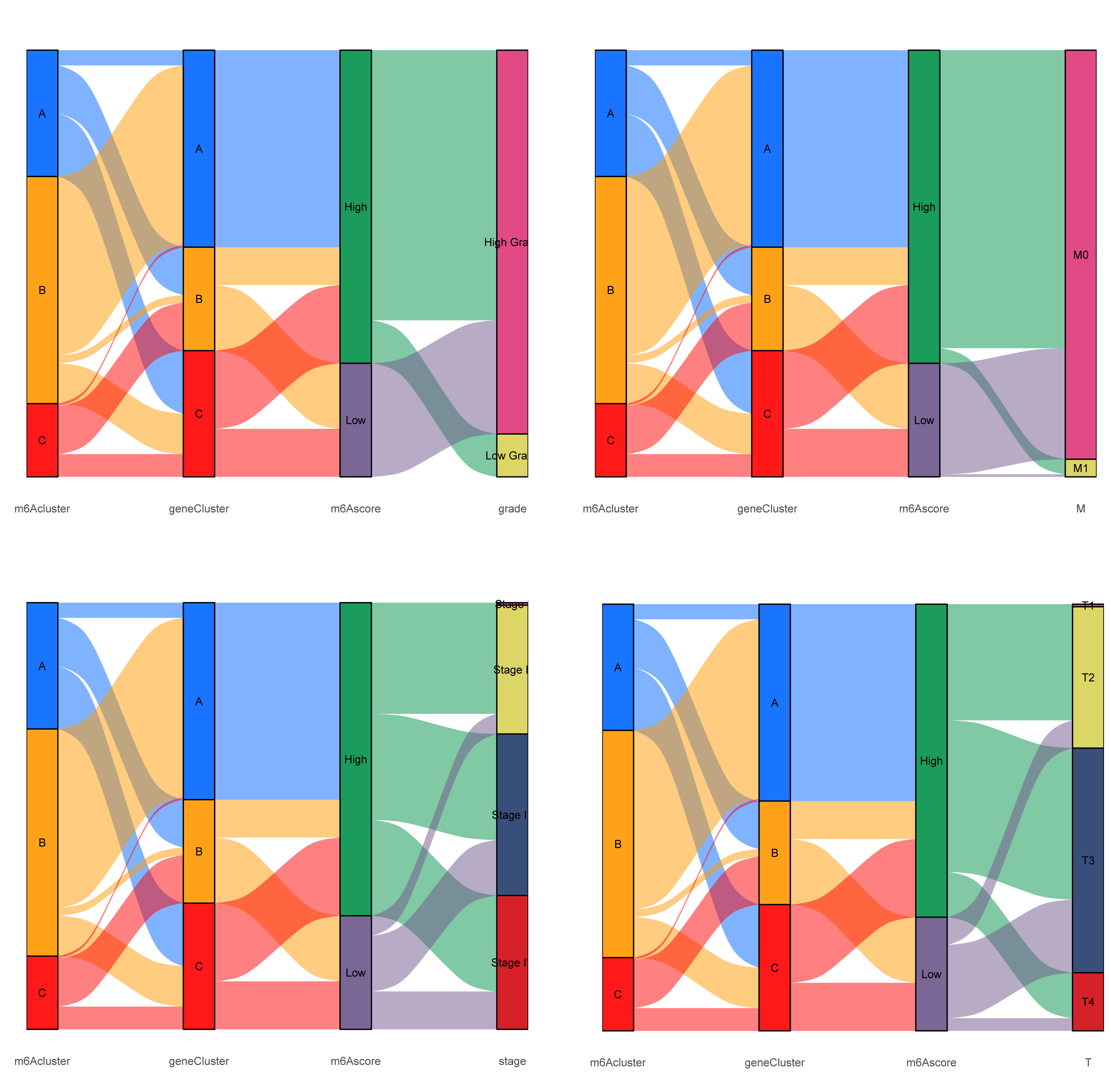

Supplement: Supplementary Figure S1 — The prognostic value of twenty-five m6a regulators were displayed via K-M survival curves. [file DataSheet_1.zip › S4.tif]
